# Wood's light as a noval diagnostic tool in aquagenic keratoderma

**DOI:** 10.1002/ski2.361

**Published:** 2024-03-12

**Authors:** Fares A. Alkhayal, Abdullah M. AlMuqrin

**Affiliations:** ^1^ Dermatology and Dermatologic Surgery Department Prince Sultan Military Medical City Riyadh Saudi Arabia; ^2^ Dermatology Prince Mohammed Medical City AlJowf Saudi Arabia

## Abstract

Aquagenic keratoderma, also known as aquagenic wrinkling of the palms, transient reactive papulotranslucent acrokeratoderma, or transient aquagenic hyper‐wrinkling, is an uncommon disorder that affects the palms and occasionally the soles. It presents with translucent whitish to yellowish papules and increased wrinkling upon water exposure. The frequency of this condition is yet to be determined among the general population. However, it is frequently observed in patients among patients with cystic fibrosis or carriers of the cystic fibrosis gene, with frequency estimated to be up to 41%. In this paper, we report the utility and novelty of Wood's light as a bedside adjunct tool to aid in diagnosing aquagenic keratoderma. To our knowledge, this is the first case reporting the use of Wood's light in diagnosing aquagenic keratoderma.

## Graphical abstract

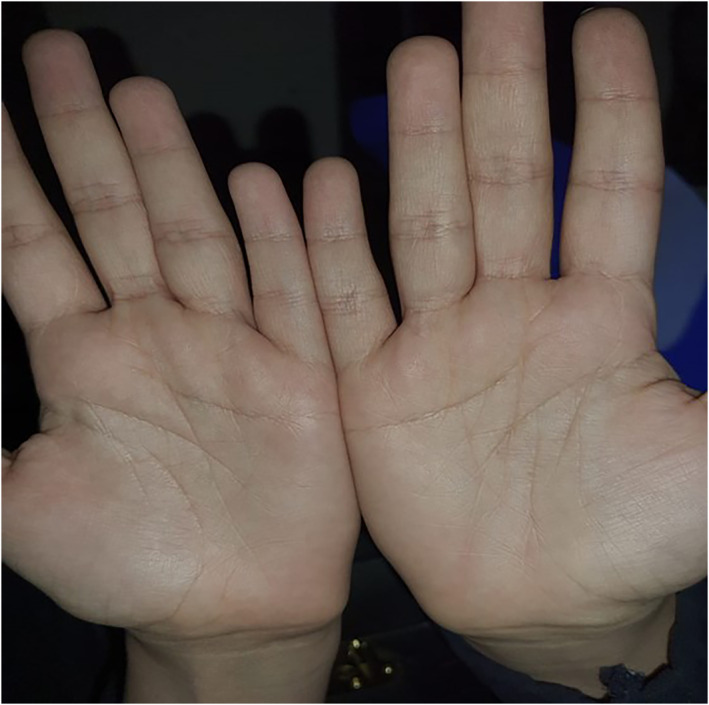

## 1 INTRODUCTION

Aquagenic keratoderma, also known as aquagenic wrinkling of the palms, transient reactive papulotranslucent acrokeratoderma, or transient aquagenic hyper‐wrinkling, is an uncommon disorder that affects the palms and occasionally the soles. It presents with translucent whitish to yellowish papules and increased wrinkling upon water exposure. It may also be associated with pruritus, tingling, and burning sensations. The frequency of this condition is yet to be determined among the general population. However, it is frequently observed in patients among patients with cystic fibrosis or carriers of the cystic fibrosis gene, with frequency estimated to be up to 41%.^1^ However, it could be sporadic or caused by medication, most frequently nonsteroidal anti‐inflammatory drugs.

In this paper, we report the utility and novelty of Wood's light as a bedside adjunct tool to aid in diagnosing aquagenic keratoderma. To our knowledge, this is the first case reporting the use of Wood's light in diagnosing aquagenic keratoderma.

## 2 CASE REPORT

A 23‐year‐old, otherwise healthy female presented to our dermatology clinic complaining of asymptomatic palm swelling and wrinkling after short contact with water. Her symptoms have only been present for a few months and occurred after exposure to water within a few minutes. The condition spontaneously resolves within almost an hour. The patient was not on any medication and her family history was negative for similar complaints.

**FIGURE 1:**
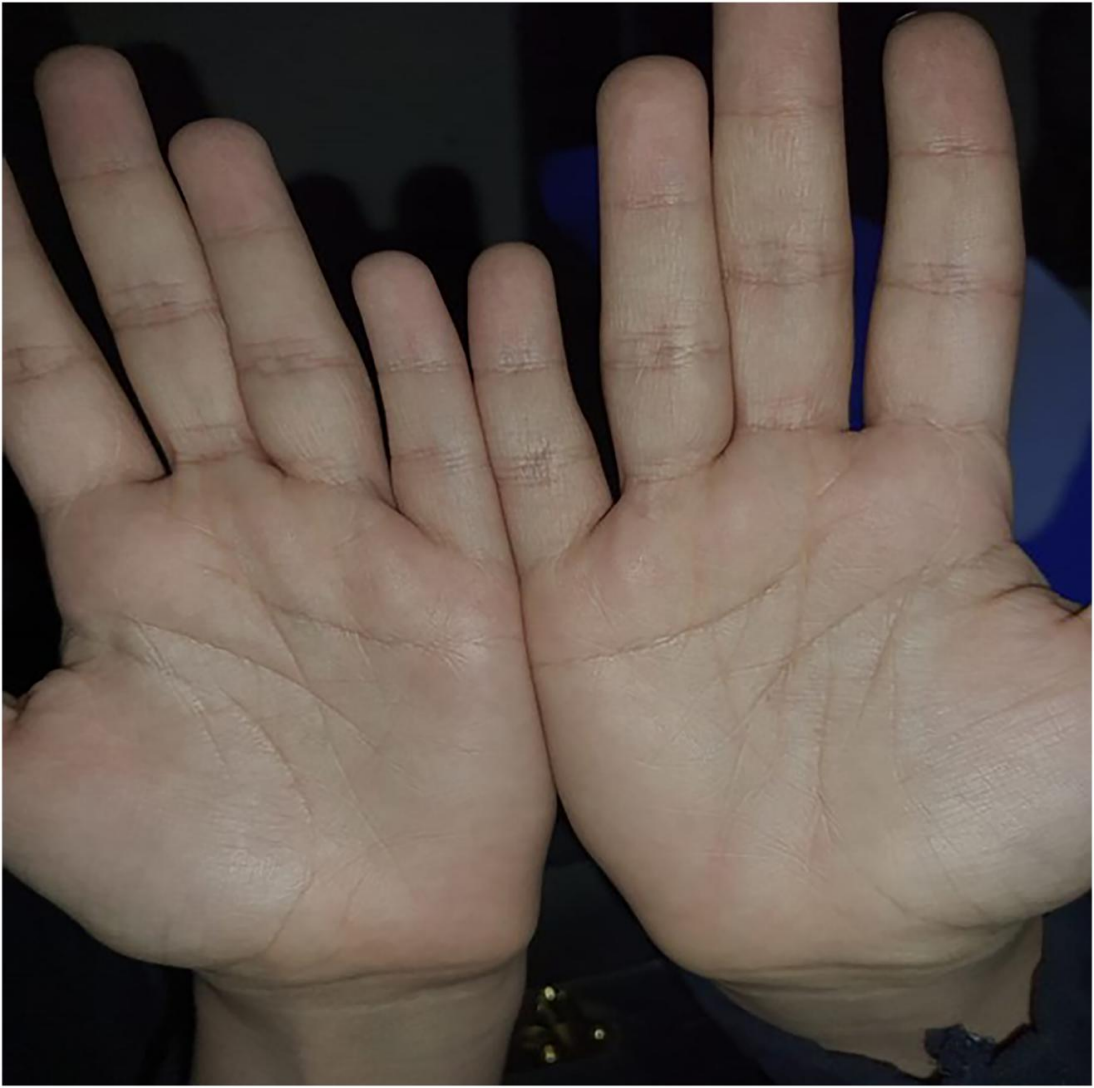
Thirty seconds after water immersion of the palms.

**FIGURE 2:**
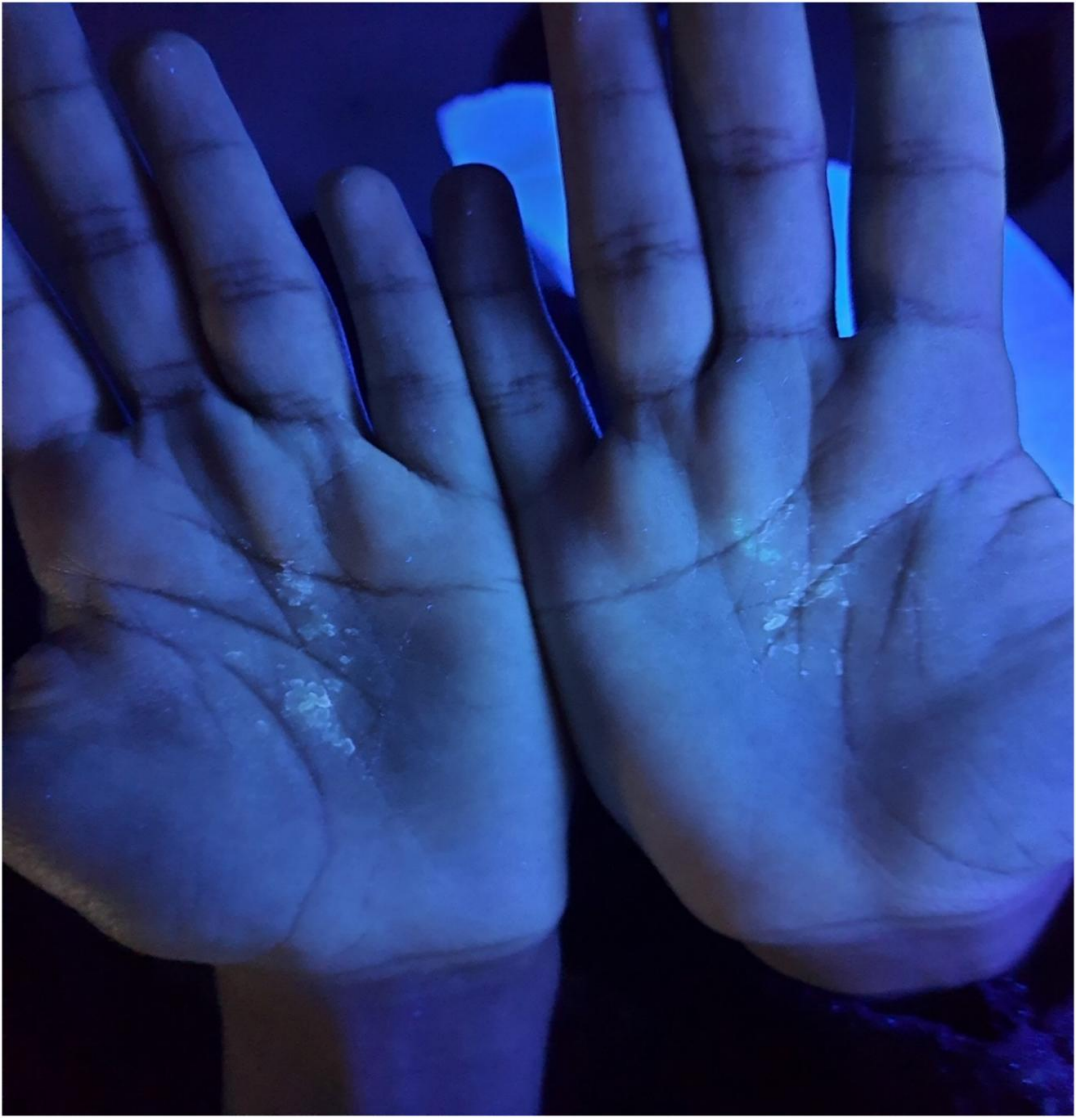
Wood's light illuminated the palms, revealing white fluorescence in less than a minute after water immersion.

**FIGURE 3:**
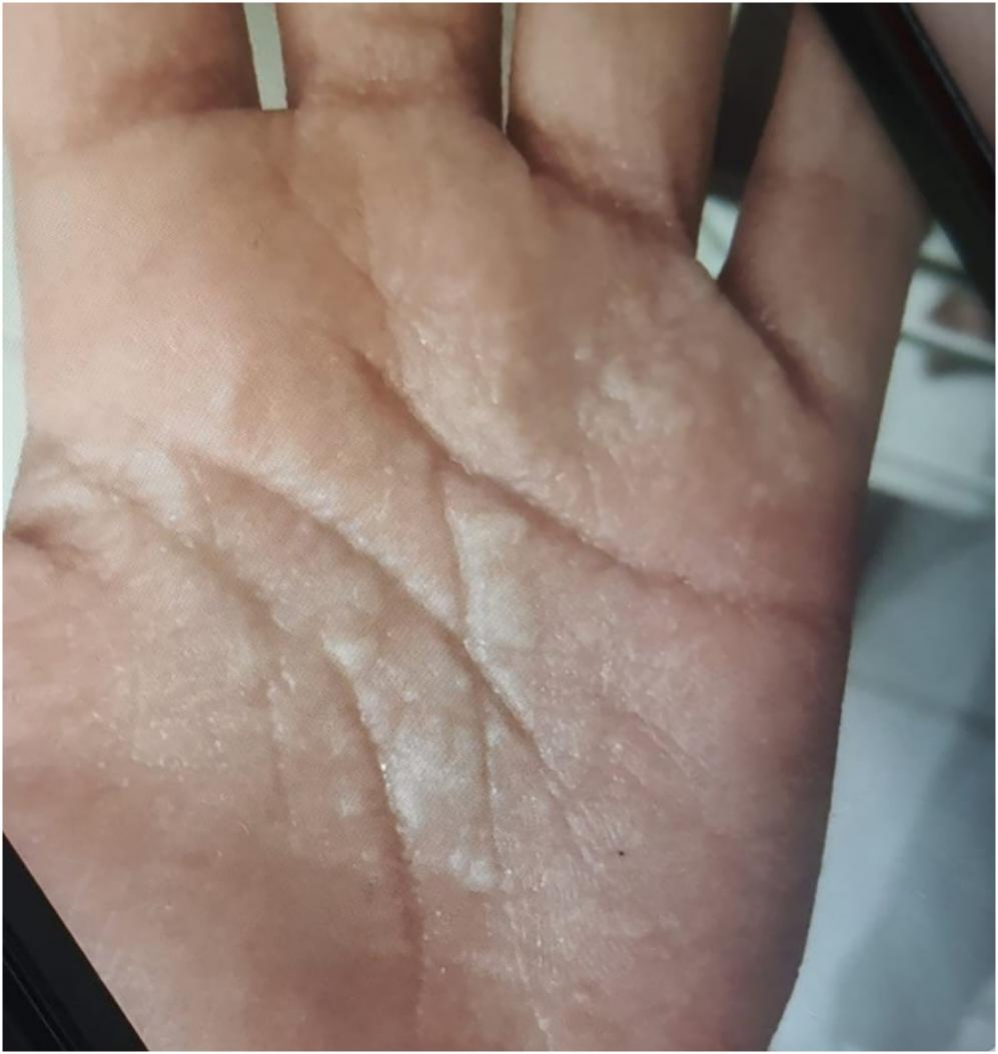
Six minutes after water immersion palms showed white papules and wrinkling.

During the patient's visit to our clinic, she was asked to wash her hands with water, and within 1 min after water exposure and before the appearance of wrinkling (Figure 1), the patient's palms were examined under Wood's light. It showed unique white fluorescence (Figure 2). Six minutes later, the patient's palm showed wrinkling and white papules (Figure 3), confirming the diagnosis of aquegnic keratoderma.

Knowing that up to 41% of aquagenic keratoderma patients have cystic fibrosis, a sweat test, which is the gold standard test for diagnosing CF, was done, and it was negative. The sweat test will be negative in Cystic fibrosis carrier individuals. The patient was treated successfully with 20% aluminium chloride and 10% urea cream. The patient's symptoms disappeared after 1 month of continued treatment application. The patient was seen 3 months later for a follow‐up, and she was clear.

## 3 DISCUSSION

The etiopathogenesis of aquagenic keratoderma is not well established. It has been postulated that the skin homoeostasis of water and electrolytes is impaired. The water channels aquaporin 3 and 5 have been shown to be dysfunctional, and that may explain the water retention seen in aquagenic keratoderma.^2^, ^3^ Among patients with cystic fibrosis, the water‐electrolytes homoeostasis impairment within the skin is viewed as a result of a mutation in the CFTR gene (*cystic fibrosis transmembrane conductance regular*), a gene responsible for transporter ion channel proteins that transport chloride and bicarbonate ions across cell membranes.^4^ CFTR protein is thought to have a functional coupling with aquaporin 3 where it imposes a triggering effect on water influx through aquaporin 3.^3^, ^4^ Furthermore, aquagenic keratoderma has been associated with some medications, including most commonly nonsteroidal anti‐inflammatory drugs, aspirin, gabapentin, and tobramycin.

Aquagenic keratoderma is a clinical diagnosis. However, a skin biopsy can be done if there is uncertainty. Common histopathological findings include dilatation of the eccrine ostia, hyperkeratosis, and hypergranulosis.^5^

Treatment of aquagenic keratoderma is often unsatisfactory. Topical treatments such as salicylic‐acid‐based products and urea‐containing creams showed variable results.^6^ However, aluminium chloride solution has been shown to dramatically improve symptoms and signs in aquagnic keratoderma.^7^ Other treatment options include injections of Botulinum toxin mainly if associated with hyperhidrosis. Medication‐induced cases can be treated with discontinuation of the culprit medication.

Non‐invasive bedside tools such as dermoscopy and reflectance confocal microscope have also been reported to be useful in establishing the diagnosis. Dermoscopic findings include three times larger eccrine ostia than the normal size.^8^ Yellow ovoid structures have also been observed as dermoscopic signs of aquagenic keratoderma.^9^ Reflectance confocal microscope was also reported to be a potentially useful non‐invasive tool in diagnosing aquagenic keratoderma.^10^

Wood's light has been used as an adjunct diagnostic tool for many dermatological disorders, including pigmentary skin disorders like vitiligo and melasma, and infectious skin diseases like erythrasma and progressive macular hypopigmentation. The fluorescence seen is the result of converting the UV light into visible light by certain molecules in the skin.^11^ Wood's light is a potentially invaluable tool revealing many characteristic fluorescence for various skin conditions if used within the appropriate context. In this case, the clinical signs of aquagenic keratoderma were not fully appreciated until Wood's light was used.

Moreover, the time needed to express the clinical signs after water exposure was less than a minute, in contrast to the 3–5 min, the classical documented time interval between the water exposure and the appearance of the clinical signs.

Wood light aids in confirming the diagnosis of aquagenic keratoderma and in ruling out other differential diagnoses including contact dermatitis or a rare form of physical urticaria (aquagenic urticaria), which occurs in response to cutaneous exposure to water and characterised by folliculocentric wheals with which flares within 20–30 min following water exposure^12^ or hereditary papulotranslucent acrokeratoderma (HPA). HPA is a rare variant of punctate palmoplantar keratoderma characterised by asymptomatic, persistent, white‐yellow translucent papules on the palms and soles and is accentuated by aqueous exposure.^13^

## 4 CONCLUSION

Wood's light is a potentially useful diagnostic tool for aquagenic keratoderma. It may also serve as a guiding tool for the skin biopsy site and therapeutic interventions such as Botox injections. We also concluded that the time interval needed to illuminate the signs of aquagenic keratoderma under Wood's lamp after water exposure is less than expected without Wood's light. There is a continuous need for further reports on aquagenic keratoderma to address different challenging aspects in diagnosing and treating this condition.

## CONFLICT OF INTEREST STATEMENT

The authors declare no conflicts of interest.

## AUTHOR CONTRIBUTIONS

**Fares A. Alkhayal**: Resources (equal); validation (equal); writing—original draft (equal); writing—review and editing (equal). **Abdullah M. AlMuqrin**: Writing—original draft (equal); writing—review and editing (equal).

## ETHICS STATEMENT

Informed consent was obtained from the participant. The participant has consented to submitting the case report to the journal.

## Data Availability

The data underlying this article will be shared on reasonable request to the corresponding author.
